# Gender Equity in Initial Teacher Training: Descriptive and Factorial Study of Students’ Conceptions in a Spanish Educational Context

**DOI:** 10.3390/ijerph19148369

**Published:** 2022-07-08

**Authors:** Laura Lucas-Palacios, Antonia García-Luque, Emilio José Delgado-Algarra

**Affiliations:** 1 Research Group of Didactics of Experimental, Social and Mathematical Sciences (DESYM), Department of Didactics of Experimental, Social and Mathematical Sciences, Area of Didactic of Social Sciences, Faculty of Education-Teacher Training Center, Campus of La Moncloa, Universidad Complutense de Madrid, 28040 Madrid, Spain; llucas03@ucm.es; 2 Research Group of Archaeological Heritage of Jaen (GIPAG), Department of Didactics of Sciences, Area of Didactic of Social Sciences, Faculty of Humanities and Educational Sciences, Universidad de Jaén, 23071 Jaén, Spain; agalu@ujaen.es; 3 Research Center on Contemporary Thought and Innovation for Social Development (COIDESO), Research Group of Didactics of Experimental, Social and Mathematical Sciences (DESYM), Department of Integrated Didactics, Area of Didactics of Social Sciences, Faculty of Education, Psychology and Sport Sciences, Campus of El Carmen, Universidad de Huelva, 21007 Huelva, Spain

**Keywords:** coeducation, feminism, higher education, teacher training

## Abstract

In recent years, progress has been made to integrate the gender perspective into university curricula. Despite this, the lack in university degrees for initial teacher training is especially remarkable. This is because, together with families, teachers have the co-responsibility of educating new generations for the future. Issues such as the low presence of concepts related to gender in teaching guides or the lack of interest of teachers regarding their inclusion in classes lead to defining research to know and understand the conceptions of a sample of 162 teachers in the initial teacher training of a Bachelor’s degree on Early Childhood and Primary Education and a Master degree of Secondary Education on the incorporation of feminist thought in education. This research is a part of a broader doctoral dissertation and a research and development project. In general terms, from a quantitative, non-experimental approach, a questionnaire is designed and sent. The analysis includes a descriptive phase and a factorial phase that allows researchers to discover latent relationships between variables. Among the main conclusions, despite showing a relatively high knowledge of feminist theory, they confuse equity and equality. Students recognize the importance of having female references with whom to identify and they consider that feminist thought is a transversal content in education.

## 1. Introduction

The national and international laws and recommendations of recent decades have led Western educational systems to eliminate the more explicit or evident discriminatory aspects against women, focusing fundamentally on the curriculum, educational materials and teacher training.

The Education 2030 Framework for Action, endorsed by the global education community in November 2015, in line with the SDG agenda, recognizes that gender equality is intimately linked to the right to education for all. Furthermore, the Framework states that achieving gender equality requires an approach that “ensures that girls, boys, women and men not only gain access to and complete different levels of education, but that they acquire the same competencies in and through education” (UNESCO 2016, p. 28). All women, girls, boys and men must have the opportunity to actively participate in society, to have their voices heard and their needs met (UN Women, 2016).

The targets of the gender goal, SDG 5, are central to achieving gender equality and women’s empowerment—a condition for achieving all SDGs, including SDG 4 on quality education for all, which itself includes a target focused on gender equality in education and SDG 8 on economic growth and decent work for all. To this end, the UN Women advocates that gender perspective integration is an essential strategy both for achieving SDG 5 and for sustainable development, and that gender equality is a crucial idea that must be made mainstream into the structures and practices of all institutions of society.

As stated in the Priority Gender Equality-Action Plan (2014–2021), gender equality refers to “the equal rights, responsibilities and opportunities of women and men, and girls and boys”. It implies that the interests, needs and priorities of both women and men are taken into consideration, recognizing the diversity of different groups of women and men. Gender equality is a fundamental human right, a necessary precondition for sustainable, people-centered development, and a goal in and of itself.

UNESCO’s vision of gender equality is in line with relevant international instruments, such as the UN Convention on the Elimination of all Forms of Discrimination against Women (CEDAW), the Beijing Declaration and Platform for Action, and the 2030 Agenda for Sustainable Development, which enshrines gender equality and the empowerment of women and girls as a stand-alone goal, Sustainable Development Goal (SDG 5), and a prerequisite for achieving the other SDGs.

For the 2030 Agenda goals to be met and for gender equality and the empowerment of girls and women to be achieved, challenges need to be addressed on the complex and cross-cutting nature of inequality: breaking down barriers that cause people, especially the most disadvantaged, to be ‘left behind’; putting in place robust and effective policies; ensuring adequate distribution of resources; achieving efficient data collection and developing monitoring and evaluation systems; and enhancing collective and inclusive action.

The impact of ideological and political interference in education in Spain has led to the approval of eight organic educational laws since 1975 with different positions on gender issues depending on party ideologies, as well as on the time and social context. As [1] pointed out, in educational matters in this country, the actual content of the law can be modified depending on the conception of education assumed by the legislator, since Article 27 of the Spanish Constitution allows substantial modifications to be made in education depending on which government is in power. Some of the latest educational reforms include shortcomings in the area of gender. Attention qualified as an attempt to be politically correct action in line with the advances and social demands, rather than a real commitment to coeducation [2].

Since the approval of Organic Law 1/2004 of December 28, on comprehensive protection measures against gender violence, and Organic Law 3/2007 of March 22, for the effective equality of women and men, all subsequent educational regulations include in their text actions aimed at achieving gender equality in the educational sphere. Thus, for example, article 24 of Organic Law 3/2007 states that the educational administrations, within the scope of their respective competences, must guarantee the equal right of women and men to education through the active integration of the principle of equal treatment in educational objectives and actions. According to this, they will develop the following actions:

1. Special attention in the curricula and at all educational stages to the principle of equality between women and men.
2. The elimination and rejection of sexist behavior and content and stereotypes that discriminate between women and men, with special consideration for this in textbooks and educational materials.
3. The integration of the study and application of the principle of equality in courses and programs for the initial and ongoing training of teachers.
4. The promotion of a balanced presence of women and men in the control and governing bodies of educational centers.
5. Cooperation with other educational administrations for the development of projects and programs aimed at fostering knowledge and dissemination, among the members of the educational community, of the principles of coeducation and effective equality between women and men.
6. The establishment of educational measures aimed at the recognition and teaching of the role of women in history.

Despite this, recent research [3,4] has highlighted the state of regression that the previous Organic Law on Education (LOMCE) entailed in terms of gender, in various aspects such as the use of sexist language in the drafting of the regulatory text, public funding to sex-segregated education, and the elimination of the subject of Education for Citizenship and Human Rights (EC hereinafter), Ethics and History of Philosophy.

The current and recent Organic Law 3/2020 of 29 December, which amends Organic Law 2/2006 of 3 May, on Education (LOMLOE), in its explanatory memorandum, states that education for society is the most suitable means to transmit and, at the same time, to renew culture, knowledge, and values that sustain it, to draw the maximum potential from its sources of wealth, fostering democratic coexistence and respect for individual differences, promoting solidarity and avoiding discrimination, with the fundamental objective of achieving the necessary social cohesion. This law adopts a gender equality approach through coeducation and promotes at all stages the learning of effective equality between men and women, the prevention of gender violence and respect for affective-sexual diversity, introducing in secondary education the educational and professional orientation of students with an inclusive and non-sexist perspective. Likewise, the Law aims to comply with the proposals in the educational field included in the Report of the Congressional Subcommittee for a State Pact on gender violence approved on 28 September 2017.

However, research that compares the current law (LOMLOE) with the previous law (LOMCE) concludes that there is progress in incorporating some norms in favor of effective equality between men and women [1]. However, the LOMLOE cannot yet be considered a feminist law, because it neglects implementation.

Another of the main focuses of attention of the laws on equality and education is the initial and ongoing training of teachers in the State Pact Against Gender Violence, in its axis 1 aimed at breaking the silence, awareness and prevention, which includes a series of measures in Section 1.1 on Education:

1. Include, in all educational stages, the prevention of gender violence, machismo and violent behavior, emotional and sexual education, and equality, also including the values of diversity and tolerance in school curricula. Guarantee their inclusion through the Educational Inspection.

9. Promote compliance with Article 7 of Organic Law 1/2004, dedicated to the initial and ongoing training of teachers, so that the requirements for the verification of official university degrees that enable the praxis of the profession of Teacher and of official university degrees that enable the praxis of the professions of Professor of Compulsory Secondary Education and Baccalaureate, Vocational Training and Language Teaching, include competencies related to equal rights and obligations of men and women, the prevention of gender violence and peaceful conflict resolution.

10. Promote compliance with Article 7 of Organic Law 1/2004, dedicated to the initial and ongoing training of teachers, so that, within the scope of teacher training schools, both in undergraduate and master’s degree studies, curricula with specialized content in pedagogies for equality and prevention of gender violence are included.

In this way, this regulation aims to address the gender training deficiencies of teachers in initial training [5,6], which are perpetuated during their continuing education through the absence of compulsory training and incentives in the vast majority of educational administrations, whose training courses are at the expense of the voluntariness of teachers with gender awareness and commitment.

The Bologna Plan already formulated the objective of reducing inequalities between women and men in Higher Education. The Organic Law 1/2004 of 28 December 2004, on Comprehensive Protection Measures against Gender Violence includes in its Title One, Chapter 4, Section 7, that universities “will include and promote in all academic areas training, teaching and research in gender equality and non-discrimination in a cross-cutting manner”. Additionally, the Organic Law 3/2007 of 22 March 2007, for the Effective Equality of Men and Women incorporates in Title II, Article 25, that it will be necessary to promote teaching and research on equality between men and women. Likewise, Organic Law 4/2007 of 12 April 2007, which amends Organic Law 6/2001 of 21 December 2001, on universities, “introduces the creation of specific programs on gender equality”, so it is remarkable the unusual or almost null presence of gender studies in the curricula of higher education despite the legal protection.

The fifth annual gender summary published by the Global Education Monitoring Report (GEM Report) highlighted that most curricula are silent on gender equality issues and that the messages conveyed by educational content can foster or undermine gender-equitable relations. It also points out that improving understanding of classroom practices is a key issue and requires further monitoring. There is also a need for more comprehensive data on gender aspects in curricula, textbooks, assessments and teacher education. Consensus also needs to be reached on what aspects of gender sensitivity in teaching practice should be included in classroom observation instruments. These efforts could benefit from being integrated into the framework of gender-responsive sector planning, as is the case with the recent collaboration between the Global Partnership for Education and the United Nations Girls’ Education Initiative (UNGEI).

In the same report, UNESCO notes that the perpetuation of gender inequality in the education system can be reduced through good quality pre-service and in-service gender training, in which teachers rethink their attitudes towards gender, as well as the perceptions and expectations of students, and learn ways to diversify their teaching and assessment styles, along the lines of the present work. Consequently, improving understanding of classroom practices is a key aspect that requires further follow-up.

However, the efforts made to integrate the gender perspective into university curricula are still very insufficient [7,8,9]. These deficiencies are especially remarkable in degrees for initial teacher training [10,11], because, together with families, teachers have the co-responsibility of educating the new generations (children and adolescents). Moreover, to this scarce presence of gender-related concepts in teaching guides must be added the lack of interest on the part of teachers, both in training and active, to incorporate them into their teaching practice [12]. Different studies [13] coincide in pointing out that active teachers perceive the inclusion of gender in their subject as unnecessary or irrelevant and that gender knowledge constitutes a secondary or peripheral topic diluted in a more general consideration of equality [9]. These resistances also present a gender bias, being greater in men than in women [5].

All the above has, as a consequence, the so-called gender blindness, which is defined by the confusion of formal equality with real and effective equality [14,15]. This situation makes it difficult to reverse gender differential socialization and break the system of mandates it establishes. In this sense, teacher training should extend beyond the conceptual, since to come to understand how their behaviors, beliefs and prejudices influence students, teachers, among other issues, should become gender aware. In this sense, the teacher is the maker of some of the most important decisions that occur in the classroom [16]. Teachers are subjective, reflective, rational subjects, who make decisions, issue judgments, have beliefs, and generate routines specific to their professional development [17,18]; moreover, their thoughts guide their behaviors [19,20].

Thus, the main objective of the present study is to analyze the connections that teachers in initial training in early childhood, primary and secondary education establish between feminist thought and coeducation to form fair and equitable citizenship. Regarding the Bachelor’s degrees in Early Childhood and Primary School Education and Master´s degree in Secondary Education, the specific objectives of this research are:

- O1: To know the level of knowledge on feminist thought of students.
- O2: To evidence the students’ conceptions of feminist thought/theory and its role in the formation of just and equitable citizenship.
- O3: To understand the educational proposals that students consider as successful to co-educate.

## 2. Materials and Methods

This study focuses on a mixed research design [21] based on positivist and interpretive paradigm approaches to know and understand the main conceptions of students of the Bachelor´s degree in Early Childhood and Primary School Education and the Master´s degree in Secondary Education (hereafter, MAES) on the incorporation of feminist thinking in education. Thus, using a questionnaire designed through Google Forms^®^, a quantitative non-experimental approach was applied. The questionnaire was distributed telematically as an instrument for the collection of information. A two-phase analysis was established: a descriptive phase and a factorial phase. The descriptive phase includes measures of central tendency, frequency and contingency tables, and the factorial phase focuses on correlations to discover latent relationships between variables [22].

### 2.1. Sample

To define the selectable population, researchers contacted university teacher-researchers who are part of the research network in Social Sciences teaching Red 14: (REF: DU2014-51720-REDT) and who have an academic and professional trajectory focused on educational innovation. About the selection of the sample, we focused on students enrolled in didactics of the social sciences [23], considering that our intention is not to generalize the results [23,24]. By limiting the population to the above-mentioned profile of students in 4 universities in the study, the sample selection was random.

**Figure 1:**
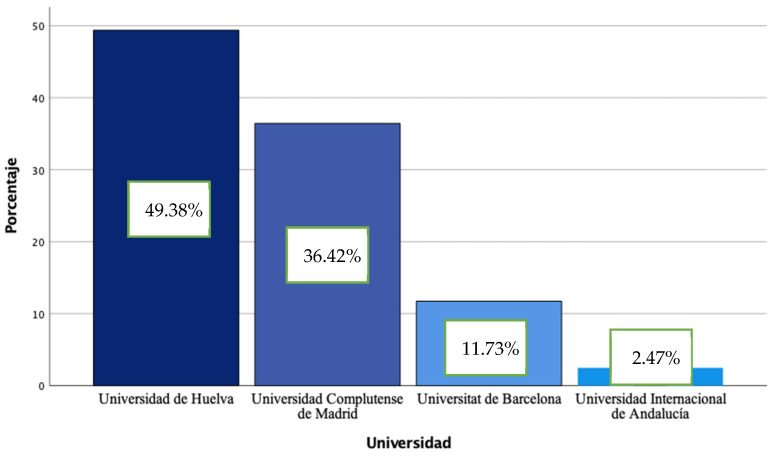
Percentage of students by participating universities.

A total of 162 teachers in initial training participated in the study; students of the Bachelor´s Degrees of Early Childhood Education, Primary Education, and MAES were from the following universities: Huelva (UHU) (*n* = 80), Barcelona (UB) (*n* = 19), Complutense of Madrid (UCM) (*n* = 59) and International of Andalusia (UNIA) (*n* = 4) (Figure 1). The number of participants in the Bachelor´s degree in Early Childhood Education was 94, 37 in the Bachelor´s degree in Primary Education and 31 in the Master´s degree in Secondary Education.

### 2.2. Instruments

Taking the theoretical framework as a reference and after reviewing the state of the art on the problem, a system of categories was developed (Table 1) based on a methodology presented in several researches [22,25]. This tool facilitates both the design of the questionnaire for data collection and the systematization of the data analysis. In this sense, within the category “feminist thought”, the first subcategory “level of initial training in feminism” is designed to know the training in gender issues that teachers in initial training have received throughout their school career. The second is “level of knowledge of feminist thought”, to check their actual knowledge of this subject. Additionally, the third is an “assessment of the contribution of feminist thought to the formation of a just and equitable citizenship”, to analyze the level of importance that teachers in initial training give to incorporating feminism in education. In this way, the collection of information is delimited.

The data collection instrument is a questionnaire designed ad hoc, constructed following a sequence of steps: (1) literature review, (2) expert appraisal and (3) piloting. The literature review includes the identification of documents related not only to the scale development procedure but also to its content. Since no instruments were identified in the literature that served our purposes, it was decided to develop a new one, taking as references the epistemological sources of the questionnaires “Citizenship and Dimensions of Memory” (CIDIMEN [Marca registrada M3695791. Titulares: Emilio José Delgado Algarra, Eloy López Meneses, Jesús Estepa Giménez y Esteban Vázquez Cano. Clasificación de Niza número 41 (Educación, Formación y Actividades Culturales)]), “Citizenship and Plurilingual Social Actor in Higher Education” (CYASPS [Marca registrada M3683892 y titular Emilio José Delgado Algarra. Clasificación de Niza número 41 (Educación, Formación y Actividades Culturales)]) and the theory of Doing Gender and other guidelines [26,27]. The final version of the instrument consists of 25 Likert-type items, 3 short-answer items (“yes”, “no”, and “I don’t know”) and 1 open-response item.

To check the reliability of the study, the correlation matrix of the 25 items of the final version of the questionnaire was inspected to eliminate those with the lowest association with the construct. As a result of the analysis, items q. 2.3, q. 2.4, q. 3.4, q. 6.4 and q. 7.1 were eliminated. Checking the compatibility of the data (KMO test and Bartlett’s sphericity, shown in Table 2) indicated that a factor analysis with the remaining 20 items was a good idea due to we obtained a value between 0.700 and 0.800 [28,29]. Moreover, the Cronbach’s alpha reliability index (Table 3) was performed. Considering that 0.700 can be considered satisfactory [30], the reliability indices 0.750 and 0.776 were obtained.

After organizing the data in SPSS, a subcategory analysis was performed: descriptive analysis and factor analysis. The descriptive study contributes to answering O1 and O2, taking as reference the frequency and the measures of central tendency: mean, mode and median. Next, we performed the correlation matrix analysis, taking into account Pearson’s correlation to address O3.

## 3. Results

**Figure 2:**
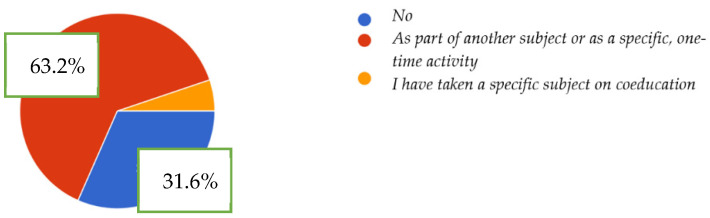
Contingency graph. Qualifications.

First objective: to know the level of knowledge of feminist thought in students Bachelor´s degrees in Early Childhood and Primary School Education and Master´s degree in Secondary Education, and according to the first subcategory, the level of initial training in feminism that our students have, and to the second subcategory, the level of knowledge of feminist thought. The descriptive statistical data show that, concerning the first subcategory, 68.4% of the students surveyed stated that they have received training in feminism or gender perspective at some point in their training. On the contrary, 51 of the 162 people surveyed (31.6%) indicated that they have never had training in gender perspective (Figure 2).

Regarding the level of real knowledge that students have of feminist theory (subcategory 2 and corresponding to questions 2, 3 and 4 of the questionnaire), we observed that when asked the question “what is feminism”, item q. 2.5 obtains high values, with a mean of 3.94 and a median of 4.00. However, the future teachers gave greater importance to item q. 2.2, with a mean of 4.59 and a median of 5.00. Based on these results (Table 4), we can say that, in general, the terms equity and equality are equated and preservice teachers do not differentiate between them. This contradiction may reflect a lack of real knowledge of feminist theory, which is why the answers to such questions are often disguised as politically correct statements.

**Figure 3:**
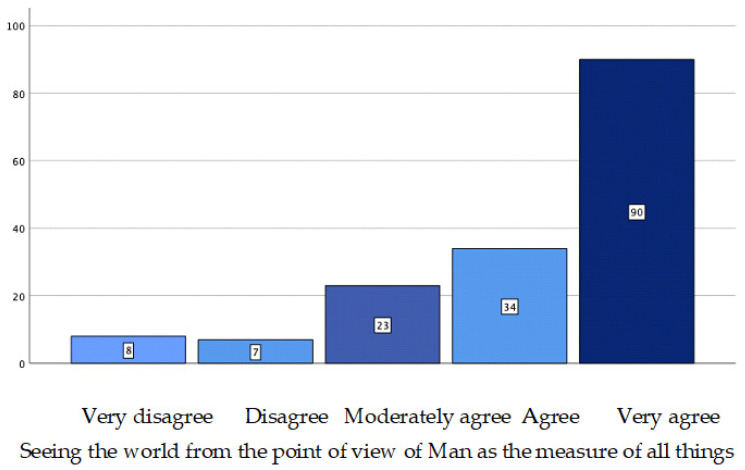
Contingency graph. Item q. 3.3.

Focusing on the question “what is androcentrism”, item q. 3.3, “to see the world from the point of view of man as the measure of all things”, is the one with the highest score, with a mean of 4.18, median of 5.00 and frequency of 90% (Figure 3). Looking at the rest of the items (Table 5), it is observed that a high and moderate agreement (mean 3.08; 3.07; 3.25) predominates in those items that define androcentrism more or less approximately (q. 3.2, q. 3.5 and q. 3.6).

In addition, in response to question 4, the results indicate that 71.6% of the respondents marked item q. 4.3, “Gender is from man to woman and domestic is what happens at home”, as correct, and only 14.8% marked item q. 4.1, “There is no difference, they are synonyms”, as correct (Table 6). This indicates that the participating students know the concepts of patriarchy and gender, as well as the relations of domination that are established between both concepts and that result in a system of violent and sexist domination in which men are shown to be superior to women in all areas of life (public and private).

**Figure 4:**
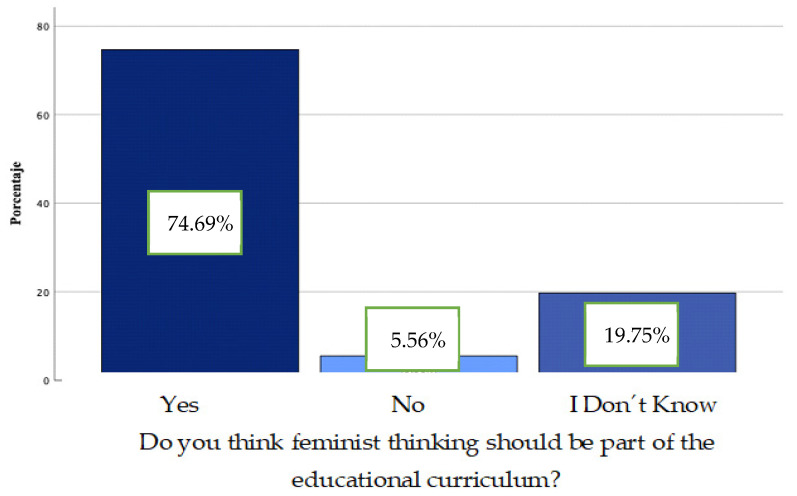
Contingency graph. Question 5.

In response to the second objective of this study, “to evidence the conceptions of the students of the Infant, Primary and MAES degrees on feminist thought/theory and its role in the formation of a just and equitable citizenship”, the percentages of question 5 revealed that 74.69% perceived the inclusion of feminism in the educational curriculum as necessary (Figure 4).

In relation to Table 7, the future teachers believe that it is positive to know feminist theory to learn citizenship (mean 3.62 and median 4.00), to make gender inequalities in today’s society visible (mean 4.28 and median 4.50) and to analyze the androcentric canon as a cause of social inequalities (mean 4.01 and median 4.00). The item on the introduction of exceptional women in classrooms and textbooks is also highly rated (mean 4.13 and median 4.00). In this sense, it is important to clarify that the simple inclusion of women in a male canon is not applying the gender perspective, because they would always seem to be included by force and their complementary and, to a certain extent, their subsidiary role would be evident. However, this type of nuance can only be recognized by people who are highly trained in the subject, which is not the case, so we can consider it a very positive result that the majority of teachers consider it important to highlight and recover the role of women throughout history.

**Figure 5:**
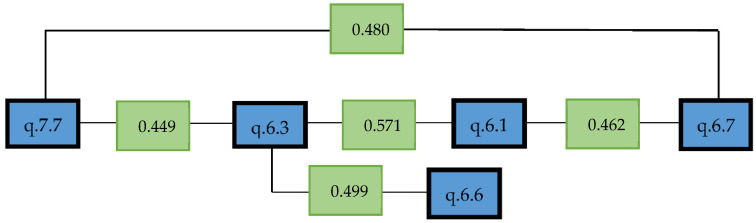
Correlational structure.

Regarding the correlations of the most outstanding items to respond to objective 3, “to see what educational proposals the students consider to be successful for coeducating”, we highlight the most significant ones (Figure 5).

q. 6.1—Integrating the feminist movement in education is the best way to learn citizenship.

q. 6.3—Feminism should be integrated in education to understand that the androcentric canon is the cause of many inequalities (gender, race, religion, etc.).

q. 6.6—Integrating the feminist movement in education makes us question the single vision of traditional history and gender as a social construction that leads to exclusion.

q. 6.7—Integrating the feminist movement in science didactics promotes a reflection of non-binary identities: Queer, LGTBI+, cis.

q. 7.7—Using heritage as a didactic tool to integrate feminist thought and to be able to reflect on gender identities as a social construction.

Item q. 7.7, which refers to using heritage as a didactic tool to integrate feminist thought and to be able to reflect on gender identities as a social construction, has a relatively high correlation (0.449 *) [* indicates than correlation is significant at the 0.05 level] with item q. 6.3, which considers that feminism should be integrated in education to understand that the androcentric canon is the cause of many inequalities (gender, race, religion, etc.). In turn, this item correlates strongly (0.571 *) with q. 6.1 (integrating the feminist movement in education is the best way to learn citizenship) (0.571 *) and with q. 6.6 (integrating the feminist movement in education makes us question the single vision of traditional history and gender as a social construction that leads to the exclusion) (0.499 *). Finally, q. 6.3. has significant correlations with q. 6.7. (integrating the feminist movement in science didactics promotes a reflection of non-binary identities: Queer, LGTBI+, cis…) (0.480 *) (0.480 *).

## 4. Discussion

The most significant results of our first specific objective, which was to know the degree of knowledge of feminist thought of teachers in initial training, reveal that a high percentage of the sample analyzed has received some kind of teaching on feminism or gender perspective throughout their training. In addition, their responses show a relatively high knowledge of feminist theory, knowing how to correctly define and detect terms such as androcentrism and gender violence. However, when feminism has to be implemented in coeducational practices, the data analyzed show that, in most cases, they confuse equity and equality. Therefore, these results are not entirely consistent with the academic literature reviewed in the field of gender, which highlights the formative shortcomings of students in faculties of education [5,6]. These results are, to say the least, striking, and even more so if we take into account that, despite knowing feminist theory, when it has to be transformed into classroom content and teaching methodologies, this didactic transposition is not always performed in the right way, or is poorly performed. Equality and equity are not the same things, and this contradiction may be a reflection of the absence of real and deep knowledge of what coeducation implies [9,15]. Most likely, future teachers know the definition of coeducation, but they have not carried out an epistemological reflection, nor are they aware of its imperative need as an educational model for the social transformation of our binary and heteronormative gender culture based on power hierarchies [5,31]. They have reached the same conclusion after investigating the perceptions and evaluations of future teachers of early childhood and primary education about a curriculum with a gender perspective. In their study, carried out with a mixed methodology in the education degrees of seven Spanish public universities (A Coruña, Alicante, Granada, Huelva, La Laguna, Malaga and Seville), they conclude that about one-third of the students, especially men, are undecided on issues related to the use of inclusive language or how coeducational criteria and evaluation systems should be.

The second objective of the study was to know the opinion of the participating students on the inclusion of feminist thought/theory to form just and equitable citizenship. All informants affirmed that it is necessary to include feminism in the educational curriculum. In addition, they consider that it is important for their future students to have feminist referents in the different fields of study to have feminist genealogies with which to identify. In addition, they state that knowing feminist theory is important to form a more just and empowered citizenry since feminism is a valuable tool and didactic content to make gender inequalities in today’s society visible and to analyze the androcentric canon as a cause of social inequalities. These results corroborate the research of [32,33], who claim that connecting the demands of feminist thought with the study of history and heritage leads to the formation of a more just and equitable citizenship. For their part, [34] show that the school constitutes the appropriate framework for the process of construction of masculinity and femininity to be possible since the division of gender roles is crucial for understanding citizenship. Along the same lines, research by [35] showed that education for citizenship with a gender focus is one of the most powerful instruments that societies have to achieve equitable change in civil society, since it leads us to analyze gender as a social construction and implies an anti-discriminatory education, education in values and respect for the other, in addition to a concession of equal opportunities.

Finally, and about the previous objective, it is worth highlighting a question that seems relevant to us and that responds to our third specific objective: to see what educational proposals are considered successful for coeducation by the students of the Bachelor’s Degrees in Early Childhood and Primary Education and the Master’s Degree in Secondary Education. We consider that an interesting finding of this research is the importance that informants give to heritage education to reflect on gender identities as a social construction and to understand that the androcentric canon is the cause of many inequalities (gender, race, religion, etc.). The fact that the majority of respondents show that feminist thinking is a cross-cutting content in education supports the research of [4,36]. One of the educational purposes is the socialization of boys and girls in their earliest stages and the formation of citizenship in more advanced stages; therefore, this is the area in which coeducation is most relevant [37]. Additionally, this is how it is perceived by the participating students, who consider that heritage is a didactic tool with great potential. Therefore, we agree with [5] in affirming that pedagogy with a gender perspective is increasingly necessary to help not only to explore and identify mismatches, but also to provide the necessary knowledge to develop gender-specific competence in the different disciplines before leaving initial university training.

## 5. Conclusions

One of the fundamental priorities of the international political agenda is to achieve real equality between women and men, as can be contemplated in the Sustainable Development Goals (SDGs). SDG 5, Gender Equality, has as one of its targets “adopt and strengthen sound policies and enforceable laws to promote gender equality and the empowerment of all women and girls at all levels”, and is aligned with SDG 4.7 (Quality Education):

Ensure that all learners acquire the knowledge and skills needed to promote sustainable development, including through education for sustainable development and sustainable lifestyles, human rights, gender equality, promotion of a culture of peace and non-violence, global citizenship and appreciation of cultural diversity and the contribution of culture to sustainable development.

Therefore, it is necessary to conduct studies that explore the incorporation of the gender approach in education and especially in teacher training processes in initial training. However, the absence of instruments to measure the degree of implementation of gender equality policy in the field of university training led us, in the first place, to develop and validate an instrument that would allow us to explore the conceptions of undergraduate and master’s degree students about feminist thought and, based on them, to be able to propose the necessary actions for the integration of feminist theory in teacher training.

According to the results shown in Section 4, the students in initial teacher training, despite acknowledging having received a large majority of training in feminist and gender issues throughout their different educational stages, confuse key and fundamental concepts such as equality and equity, although there are others, such as gender and patriarchy, whose epistemological understanding is high. However, their conceptual knowledge does not necessarily imply the acquisition of gender awareness, transforming commitment and coeducational professional competencies. Given this reality, it is necessary to make a critical analysis of the type of training that is being carried out (where, how, when, by whom and from what perspective) since it is causing not only a conceptual confusion but also a devaluation of feminist theory that can lead to resistance to education in and for gender equality. Therefore, we consider it important that initial teacher training on feminist issues should extend beyond conceptual learning or methodological learning merely aimed at the implementation of strategies to work in the classroom activities without sexist biases. Teachers must acquire gender awareness to understand that their behaviors, beliefs and prejudices have a determining influence on their students, whether positive or negative. Likewise, this training must be carried out by trained, committed and qualified professionals, and must not respond to the voluntarism of feminist ideology personnel who, with the best of their intentions, incorporate the gender perspective in their subjects, often erratically due to lack of training, since goodwill and interest are not enough to exercise a coeducational teaching praxis.

On the other hand, and taking into account the results obtained, we must also highlight the importance of heritage as a key to identity (social, individual and gender), being an essential educational tool to achieve the strengthening and social cohesion and to strengthen self-confidence and proper human development.

Finally, we should point out the limitations of this study. The first limitation refers to the sample, specifically to its size and origin: although the sample was collected from different university training institutions in different Spanish universities, obtaining a larger sample size and extending the collection to other autonomous communities would contribute to its representativeness and the generalization of the results. On the other hand, it would be interesting to complete the collection of information with other instruments, such as interviews or discussion groups, and even to contrast the results with the vision of active teachers, to have a more complete vision and to be able to detect possible gaps that could be filled in the training of future teachers.

Even so, in this research, we offered some guidelines for incorporating the gender perspective in formal education:

- Incorporate compulsory subjects on gender and equality issues in study plans, as required by the current regulations (State Pact against Gender Violence), and not as an optional or free configuration subject, which is the most frequent.
- To offer specific degrees in gender and equality in the educational offer of Degrees, which extend beyond the Masters, Expert Degrees, Doctorate Programs and lines within some doctorate programs.
- Offering complimentary training courses. In this case, each university organizes its complementary training actions for students, faculty and administrative and service staff. The handicap of this type of training is precisely its optional nature, so their success depends on the incentivized obligatory nature.
- Incorporate the gender perspective in the transversal competencies of the curricula, or some of the contents of some of the subjects.
- To offer teachers in faculties of education quality feminist training so that they can not only incorporate this perspective in their teaching, regardless of whether it is a specific topic of the subject or whether it is (or not) included in the transversal competences of the subject they teach, but also to awaken gender awareness and commitment to transformation.

We conclude by saying that it is essential to continue researching and offering new perspectives and approaches to gender analysis in teacher training to provide both active teachers and future teachers with tools to educate in equality (coeducation), and for the school to be an institution where the formation of critical and egalitarian citizenship is learned and developed. Therefore, we believe that this study can be the initial step toward an action–research project focused on increasing gender awareness in the university training of future teachers through heritage education, to analyze, validate and implement it in Spanish faculties of education.

## Figures and Tables

**Table 1:** Categories, subcategories and descriptors of the study. Source: own elaboration.

Categories	Subcategories	Descriptors	Items
Feminist Thought	Level of initial training in feminism	Students without gender training	q. 1.1, q. 1.2, q. 1.3
		Students who report having received gender training in a cross-cutting manner or as part of some other subject.	
		Students who have taken a specific gender subject.	
	Level of knowledge of feminist thought	Consideration of feminism as a street movement and unrelated to its theoretical and scientific contributions. Androcentric vision.	q. 2.1, q. 2.2, q. 2.3, q. 2.4, q. 2.5, q. 3.1, q. 3.2, q. 3.3, q. 3.4, q. 3.5, q. 3.6, q. 4.1, q. 4.2, q. 4.3
		Superficial knowledge of sex-gender theories, gender roles, identities, and feminine–masculine binary approach.	
		Gender as a school or perspective that questions the approaches of traditional history and its androcentric character.	
		Gender in its social dimension, as an instrument that allows us to better describe or change reality.	
	Assessment of the contribution of feminist thought to the formation of just and equitable citizenship.	Androcentric vision (account of important men).	q. 5.1, q. 5.2, q. 5.3, q. 6.1, q. 6.2, q. 6.3, q. 6.4, q. 6.5, q. 6.6, q. 6.7, q. 7.1, q. 7.2, q. 7.3, q. 7.4, q. 7.5, q.7.6, q. 7.7
		Presents only exceptional women as an addiction to the androcentric narrative. Differential social consideration of women.	
		We do not necessarily have to know the works achieved by women, but we do have to question the unique vision of traditional history and gender as a social construction that leads to exclusion.	
		It presents a vision of feminist theories under the prism of critical didactics. It promotes a reflection on non-binary identities: Queer, LGTBI+, cis.	

**Table 2:** KMO test and Barlett´s sphericity.

**Kaiser–Meyer–Olkin Measure of Sampling Adequacy**		0.750
**Bartlett’s test of sphericity**	Approx. chi-squared	919.761
	gl	190
	Sig.	<0.001

**Table 3:** Cronbach’s alpha test.

Reliability Statistics		
Cronbach’s Alpha	Cronbach’s Alpha Is Based on Standardized Items	Number of Items
0.758	0.776	20

**Table 4:** Descriptive statistics of central tendency for question 2.

		The Opposite of Machismo	Doctrine and Social Movement that Seeks Equal Rights between Men and Women	Women Who Hate Men	A Social Movement That Seeks the Superiority of Women in the Face of Patriarchy	A Political Movement That Seeks Equal Rights for Women and Men
N	Valid	162	162	162	162	162
	Lost	0	0	0	0	0
Mean		1.56	4.59	1.20	1.40	3.93
Median		1.00	5.00	1.00	1.00	4.00
Mode		1	5	1	1	5
Deviation		1.069	0.923	0.588	0.888	1.307

**Table 5:** Descriptive statistics of central tendency for question 3.

		Seeing the World in a Sexist Way	Seeing the World from the Point of View of Men as the Measure of All Things	Androcentrism Does Not Exist	Social Thinking That Attributes Different Social Characteristics to Each Sex	Thinking That Naturalizes Gender Roles and Stereotypes
N	Valid	162	162	162	162	162
	Lost	0	0	0	0	0
Mean		3.08	4.18	1.56	3.07	3.25
Median		3.00	5.00	1.00	3.00	3.00
Deviation		1.392	1.136	0.870	1.315	1.281
Sum		499	677	252	497	526

**Table 6:** Descriptive statistics of the frequencies of question 4.

		Frequency	Percentage	Valid Percentage	Accumulated Percentage
**Valid**		**1**	**6**	**6**	**6**
	There is no difference; they are synonyms	24	14.8	14.8	15.4
	Domestic is the one that happens at home, such as gender violence	21	13.0	13.0	28.4
	Gender is from the man to the woman and domestic is the one that happens at home	116	71.6	71.6	100.0
		162	100	100.0	

**Table 7:** Measures of key trends (mode, median and arithmetical average) for question 6.

		It Is the Best Way to Learn Citizenship through Knowledge of Its Theory, Characteristics and Historical Evolution	It Is an Effective Didactic Tool to Visualize Gender Inequalities in Today’s Society	It Is an Effective Didactic Tool to Analyze the Androcentric Canon as a Cause of Social Inequalities (Not Only Gender Inequalities but Also Race, Religion, Etc.)	I Do Not Think It Contributes Anything nor Should It Do So	The Introduction of Exceptional Women in Classrooms and in Textbooks	It Makes Us Question the Single Vision of Traditional History and Gender as a Social Construction That Leads to Exclusion	It Promotes a Reflection of Non-Binary Identities: Queer, Lgtbi+, Cis
N	Valid	162	162	162	162	162	162	162
	Lost	0	0	0	0	0	0	0
Mean		3.62	4.21	4.01	1.31	4.13	3.91	3.75
Median		4.00	4.50	4.00	1.00	4.00	4.00	4.00
Desviation		1.064	0.921	1.019	142	1.051	1.213	1.192
Sum		516	693	649	223	669	634	607
